# Why Knowledge Sharing in Scientific Research Teams Is Difficult to Sustain: An Interpretation From the Interactive Perspective of Knowledge Hiding Behavior

**DOI:** 10.3389/fpsyg.2020.537833

**Published:** 2020-12-08

**Authors:** Feng Liu, Yuduo Lu, Peng Wang

**Affiliations:** ^1^School of Economics and Management, Dalian University of Technology, Dalian, China; ^2^Institute of China Innovation and Entrepreneurship Education, Wenzhou Medical University, Wenzhou, China

**Keywords:** sustainable development, knowledge sharing, knowledge hiding, behavioral interaction, research teams

## Abstract

Efficient knowledge sharing is an important support for the continuous innovation and sustainable development of scientific research teams. However, in realistic management situations, the knowledge sharing of scientific research teams always appears to be unsustainable, and the reasons for this are the subject of considerable debate. In this study, an attempt was made to explore the interactive mechanism of knowledge hiding behaviors in scientific research teams between individual and collective knowledge hiding behaviors and its impact on knowledge sharing by adopting grounded theory to comprehensively understand this situation. The results show that knowledge hiding behavior in the scientific research team is a two-phase interactive process and is capable of affecting sustainable knowledge sharing by reducing the supply of knowledge, creating a poor knowledge sharing atmosphere, and forming an interpersonal distrust relationship. This research may provide a strong basis for a deeper understanding of the interaction mechanism of knowledge hiding behavior and its impact on knowledge sharing.

## Introduction

Currently, human society has spanned from the era of the industrial economy to the era of the knowledge economy (Kim and Mauborgne, 1998). The creation, dissemination, and use of knowledge, information, and data are restructuring traditional economic development forms into knowledge-based development. Knowledge has become an “intangible asset” that promotes sustainable development and continuous innovation in various organizations (Grunwald, 2004). In this macro background, the scientific research team, as an important organizational form, is facing increasing competition and assessment pressures and demands for innovation (Peltokorpi and Hasu, 2016). Notably, knowledge has become the core resource, which has gradually replaced traditional scientific research funds, equipment, and venues, for the survival and competition of the current scientific research teams. The sustainable development of scientific research teams and the output of high-quality scientific research achievements are inseparable from the continuous acquisition of new knowledge and efficient knowledge utilization in the knowledge economy (Johnson, 2017). Therefore, how to continuously acquire new knowledge and effectively use the existing knowledge mastered by scientific research teams has become the core issue and key practical problem that the knowledge management of the scientific team must address (Gloet, 2006). Relevant research results have shown that effective knowledge acquisition and utilization within the scientific research team are the key to restricting the success of implementing knowledge management and further verifying that good knowledge sharing among scientific research team members, which plays an important role in scientific decision-making, improves the effectiveness and efficiency of the scientific team’s knowledge management. Therefore, knowledge sharing among members of the scientific research team can inevitably lead to a significant increase in the efficiency of the scientific research team in using and creating knowledge. Based on this, the scientific research team can promote knowledge flow, sharing, and collaborative knowledge creation among knowledge workers of the scientific research team by specifying appropriate knowledge sharing incentive strategies, which can lay a strong foundation for the sustainable development of the scientific research team (Zhuge, 2002; Sung and Choi, 2012).

However, in practical knowledge sharing scenarios in scientific research teams, one of the most common behaviors, namely, knowledge hiding, is widespread and makes it difficult to sustain knowledge sharing within scientific research teams (Huo et al., 2016). Existing research on knowledge hiding has investigated its antecedents and consequences, but the exploration and interpretation of the characteristics and the interaction between the targets and the perpetrators of knowledge hiding are still limited (Connelly and Zweig, 2015; Connelly et al., 2019). From this point of view, Connelly and Zweig (2015) pointed out that knowledge hiding behavior can be a mutual influence between targets and perpetrators. Zhao et al. (2019) proved that leader–member exchange (LMX) may affect how much they hide knowledge from their colleagues. These studies reveal that knowledge hiding behavior interacts among individuals. However, it is still unclear whether this interaction exists between individuals and teams. In other words, the exact interaction mechanisms of knowledge hiding behavior in organizations or research teams are still unclear.

Therefore, the first aim of this study is to explore (1) the interaction mechanism of the individual and the collective knowledge hiding behavior. In addition, considering the full range of the outcomes of knowledge hiding has yet to be examined (Connelly et al., 2019), such as how knowledge hiding and its interaction between individuals and the team affect knowledge sharing. The second aim of this paper is to reveal (2) the relationship between knowledge hiding and knowledge sharing within scientific research teams.

Our theoretical views will make significant contributions to research on knowledge hiding and knowledge sharing. First, we expanded the research on knowledge hiding behavior from the traditional dyadic level to the collective level and constructed a cross-level interactive cycle model between individual knowledge hiding and collective knowledge hiding in research teams, which deepened the academic community’s understanding of hierarchical interaction on knowledge hiding behavior and broadened the perspective of knowledge hiding behavior research. Second, we identified two new influencing factors that affect the relationship between knowledge hiding behavior and knowledge sharing: reducing knowledge supply and forming a poor knowledge sharing atmosphere. These findings enrich the research on the antecedent factors of knowledge sharing and deepen the academic and practical understanding of the relationship between knowledge hiding and knowledge sharing. Moreover, from a practical point of view, our research helps explain how knowledge hiding behavior forms cross-level interactions, which in turn affects knowledge sharing within the research team and what management measures can be used to solve these problems.

## Literature Review

### Knowledge Sharing

Existing studies on knowledge sharing have discussed from multiple dimensions and formed two different research paradigms. The first research paradigm is a technology-centric paradigm (McElroy, 2000). It is characterized by technology as an important factor for knowledge sharing. This paradigm can be further divided into the communication and tool perspectives. From the perspective of communication, scholars believe that knowledge sharing is an effective way of interaction and communication (Mei et al., 2004) and can also be enhanced by the communities of practices (Manuti et al., 2017). Active communication between knowledge holders and receivers contributes to knowledge sharing, leading to the effective management of knowledge within the organization. From the tool perspective, knowledge management researchers pay significant attention to the form of knowledge sharing media and the impact of knowledge sharing, such as the impact of IT technology, practical seminars, and teams on knowledge sharing (Choi et al., 2010). The second research paradigm is based on the people-centric paradigm, that is, the “interpersonal interaction” and “interpersonal relations” as entry points, focusing on the role of interpersonal structure and relationships on knowledge sharing. This paradigm can be further refined into an interactive perspective, learning perspective, power perspective, market perspective, and social exchange perspective. The interactive perspective was represented by Japanese scholars, such as Nonaka and Takeuchi (1995), focusing on exploring the interaction between tacit and explicit knowledge. The learning perspective focuses on the connotation of knowledge sharing from the perspective of individual learning and development, team learning, collective development (Zellmer-Bruhn and Gibson, 2006; Manuti et al., 2017), and the learning climate/culture (Watkins and Marsick, 2003). The power perspective is characterized by team members’ ownership of private knowledge, which is regarded as a kind of power resource, and the process of re-sharing private knowledge is also a process of allocating power relations among people (Robert et al., 2009). Furthermore, the market perspective defines knowledge as a unique, exclusive resource that can be bought, sold, and exchanged in the research team (Nonaka and von Krogh, 2009). The social exchange perspective regards knowledge sharing as an exchange (Liu et al., 2011). Under these two paradigms, scholars have carried out extensive discussions with the objective of promoting knowledge sharing; however, the discussion on the obstacles to knowledge sharing is relatively limited (Connelly et al., 2012). Therefore, it is necessary to appropriately improve the current discussion from the perspective of obstacles to knowledge sharing. Considering that although knowledge hiding behavior is not necessarily a kind of behavior that harms the organization’s knowledge sharing, to a certain extent, its impact on the sustainability of knowledge sharing is obvious. Therefore, exploration of the obstacles to knowledge sharing from the perspective of knowledge hiding behavior is desirable.

### Knowledge Hiding Behavior

In 2012, the concept of knowledge hiding behavior was proposed to describe an intentional attempt by an individual to withhold or conceal knowledge that has been requested by another person (Connelly et al., 2012). Once the knowledge hiding behavior was proposed, it immediately attracted significant attention of scholars in the field of knowledge management and organizational behavior. At present, the current research on knowledge hiding behavior mainly focuses on its antecedents and consequences (Connelly et al., 2019). From the perspective of antecedents, previous studies have shown that knowledge hiding behavior is affected by many different factors, such as intellectual psychological ownership and territorial behavior (Peng, 2013), complexity of knowledge, relevance of knowledge and tasks (Connelly et al., 2012), high distrust and competitiveness (Hernaus et al., 2019), dark triad psychological traits (Pan et al., 2018) and Big Five personality (Anand and Jain, 2014), task interdependence (Gagné et al., 2019), LMX (Zhao et al., 2019), and performance-proven goal orientation (Zhu et al., 2019). From the perspective of its consequences, studies have shown that knowledge hiding behavior can cause greater interpersonal distrust (Connelly et al., 2012), the deterioration of interpersonal relationships (Connelly and Zweig, 2015), the reduction individual and team creativity (Bogilović et al., 2017; Rhee and Choi, 2017), the reduction of psychological safety (Jiang et al., 2019), and so forth. Although these studies provide important information to understand knowledge hiding behavior, most of the published articles focus on dyadic levels. As suggested by Černe et al. (2015), it is clear that research on knowledge hiding behavior need not only focused on dyadic levels but also on collective knowledge hiding behavior. In addition, research focusing on the difference and interaction mechanism between individual and collective knowledge hiding behaviors within scientific research teams has not yet been adequately discovered, and research on how this behavioral interaction affects knowledge sharing requires further improvement. Therefore, it is necessary to explore the interaction mechanism from the perspective of the social interaction of knowledge hiding behavior and further interpret the impact on knowledge sharing. Specifically, it is valuable to explore how the knowledge hiding behavior of individual members within the scientific research team is transferred to the scientific research team, and how the collective knowledge hiding behavior of the scientific research team affects the individual members. Further, undeniably, more systematic explorations are still required to reveal the impact of this interaction on the knowledge sharing of scientific research teams, which has important theoretical and practical value for supplementing and improving research on knowledge hiding behavior and knowledge sharing.

## Research Design

### Research Method

The qualitative method was used because the main objectives of the study were to answer “how and why” questions. In addition, qualitative methods allow for an overall understanding of the complex phenomenon under investigation by allowing researchers to carry out an empirical inquiry that investigates a bounded contemporary phenomenon within a real-life context (Creswell, 2013). Furthermore, among the qualitative research methods, grounded theory is considered to be one of the most important qualitative research methods in the field of philosophy and social sciences because of its special research question presentation methods and rigorous data analysis methods (Corbin and Strauss, 1990; Strauss and Corbin, 1997). In recent years, the grounded theory research method has been widely used in the field of organizational behavior and knowledge management research (Benoliel, 1996; Danielsson et al., 2019), which provides good support for the use of grounded theory in our study. Furthermore, related research also noted that the grounded theory research method is, in particular, suitable for analyzing micro-behavior and social interaction processes, which is mostly in line with what we are concerned with. Therefore, this study utilized the grounded theory research method to explore the interaction mechanism of knowledge hiding behaviors in scientific research teams and carried out further research (McCann and Polacsek, 2018).

Thus far, grounded theory has been classified into three main types that are connected yet different: the original version of the grounded theory originally proposed by Glaser and Strauss (1967), that is, the classic (original) grounded theory, the proceduralized version, and the constructivist’s approach to grounded theory (Charmaz, 1996; Strauss and Corbin, 1997). There are some differences among the three schools with respect to the process of epistemology and coding. The proceduralized version based on hermeneutics is more suitable for this study. This is not only because this research paradigm is the most widely used, but also because the proceduralized version of grounded theory provides a standardized analysis technique that will play an important role in analyzing and predicting specific behaviors. Based on the above-mentioned discussion, this research follows the research paradigm of the proceduralized version of grounded theory to guide the corresponding qualitative data collection and analysis.

### Sampling and Research Sample Selection

In grounded theory research, the following three common sampling methods are involved: theoretical sampling, objective sampling, and selective sampling (Sandelowski, 1995; Robinson, 2014). Among them, theoretical sampling is known to develop the theory. Researchers are often unsure who the next sample is during the research process, and the sampling object is entirely driven by the theory. In contrast, objective sampling is known for selecting rich case data and conducting in-depth research. In objective sampling, researchers can identify in-depth events to achieve in-depth discussions on research-related issues. Selective sampling can solve several problems, such as researchers’ time constraints and research frame limitations, and can enhance the feasibility of research. Based on the research objective of this study, as well as the feasibility and convenience of the study, objective and selective sampling were selected. That is, scientific research teams and members who are interested in the interaction of knowledge hiding behaviors and have the time and experience to provide the most detailed information on knowledge hiding behaviors were selected as the sampling objects of this research. Finally, through classroom recruitment, friend introduction, and active visits, interactive interview information was collected on the knowledge hiding behaviors of 31 research team members in 9 research teams from different disciplines, including innovation management, history, chemistry, and molecular biology. We chose participants who were nested in teams, since we needed to analyze the collective knowledge hiding behavior at the team (collective) level. In this way, the triangle verification of the discourse among different members of the same team ensures the reliability and validity of data collection at the team level.

The interviewees were mainly in the age range of 24 to 56, including 22 males and 9 females. The interviewees’ work experience was between 0 and 28 years. All respondents had bachelor’s degrees and 14 had doctorates. In order to effectively implement triangular verification of the data, the study also collected the work diaries of some employees to supplement and verify the interview data. In addition, for interviewees, they were compensated with a gift that was worth 200 RMB.

### Collection of Qualitative Research Data

Semi-structured, open, face-to-face interviews were primarily conducted to collect data on the interaction mechanism of the cross-level knowledge hiding behavior of scientific research teams and their impact on knowledge sharing. Interviews are one of the most commonly used research methods in qualitative research, and face-to-face interview is the most commonly used research method among interview methods (Gillham, 2000). Moreover, face-to-face interview can also enable researchers to capture variation of details in the facial expressions of the interviewees, as well as sound performance and body movements, which can provide relevant information for grounded theory research. This research focuses on the cross-level interaction of knowledge hiding behavior within scientific research teams and its impact on knowledge sharing, which needs to fully collect the ideas of scientific team members when making interactive decisions on knowledge hiding behavior. Therefore, semi-structured, open, face-to-face interviews are more suitable for this research. In order to improve the efficiency of the interviews, we established the outline of an interview, as presented in Table 1. The interview themes mainly focused on “the *status quo* of knowledge hiding behavior of scientific research team members,” “the interactive process and mechanism of knowledge hiding behavior in scientific research teams,” “the intervention of knowledge hiding behavior in scientific research teams,” and “the impact of knowledge hiding behavior interactions on knowledge sharing in scientific research teams.”

**TABLE 1:** Outline of an interview.

Interview theme	Main content
The *status quo* of knowledge hiding behavior of scientific research team members	Is there knowledge hiding behavior among your colleagues? How about the frequency? How did they do?
	Have you ever experienced knowledge hiding? How did you do it? Why?
The interactive process and mechanism of knowledge hiding behavior in scientific research teams	If someone in your team engages in knowledge hiding behavior, do you think that this behavior will affect the organization and you personally? Why?
	If everyone in your team hides their knowledge, what would you do?Why?
Intervention of knowledge hiding behavior in scientific research teams	What factors do you think might promote or inhibit the hidden knowledge of team members?
	If the organization wants to encourage or suppress knowledge hiding within the team, what should organization do and why?
The impact of knowledge hiding behavior interactions on knowledge sharing in scientific research teams	Do you think that knowledge hiding has an impact on sustainable knowledge sharing, and why?

Considering that the respondents of the knowledge hiding behavior of the scientific research team may perceive social desirability (Furnham, 1986) as a relatively negative concept, knowledge hiding behavior may cause social desirability when a third person is involved. In order to protect the participants and obtain interview data with good reliability and validity, face-to-face interviews were conducted and completed by the authors of this article alone. The authors communicated with the interviewees about the interview themes in advance via telephone or *WeChat*. In the actual implementation of the interview, the interview was carried out according to the outline but was not limited to the outline. The interview was generally selected by the interviewee. Usually, a quieter, independent office was selected. In addition, the authors protected the information of each interviewee, and it would not be mentioned by any third person. Different interviewees within the same team were also independent of each other. The average interview time length was 47 min. Finally, when 31 members of the scientific research team were interviewed, 82 pages of transcripts were created. The data and theory became saturated, and we stopped collecting the interview data.

## Research Data Analysis

After data collection, we entered the data analysis stage. As mentioned above, we drew on the analysis thought of the proceduralized version of grounded theory, which includes open, axial, and selective coding for our data analysis. It is important to mention that Excel was used to organize the coding and to better observe the relationship between different categories.

### Open Coding

Open coding was the first step in our study, and it is a process of analyzing interview data word by word and sentence by sentence, and refining meaningful concepts (Pieterse, 2011). This study followed the principle of “live coding,” which involved the extraction of as much of the interviewee’s original words as possible. In our study, two authors independently coded the interview data. After the authors’ independent coding, 624 “live codings” were obtained. Subsequently, the authors merged the same codes, discussed and determined the different codes together, and finally formed the relevant “live coding,” in this case, about 500, for further categorization. The categorization process is a process of sorting and categorizing concepts. Considering the disciplinary attributes of research, standard concepts in the fields of management and organizational behavior were selected to represent “live coding.” Table 2 presents the results of the categorization in this research. According to Table 2, the main concepts included in this study mainly comprise individual knowledge hiding behavior, collective knowledge hiding behavior, individual status, work interdependence, herd mentality, imitative learning, collectivist orientation, team identification, leaders’ supervision, purpose of assessment, poor knowledge sharing atmosphere, reduced knowledge supply, interpersonal distrust, and knowledge sharing.

**TABLE 2:** Results of categorization.

Source sentence: conceptualization	Categories
How does the behavior occur? Usually because one person hides knowledge first. Someone did this, that is to say, he/she would hide what he/she knew so I would learn from him/her. If everyone does not hide, I believe a few people will hide. However, when an individual breaks the rules and hides what he/she knows, it is likely to cause chain reactions.	Individual knowledge hiding behavior
When everyone hides knowledge, we basically form a culture, and an individual behavior becomes a collective behavior. For example, if we all feel that our group leader is a person who does not like to share, everyone in our group will gradually hide their knowledge. In my opinion, I think team-level knowledge hiding is widespread.	Collective knowledge hiding behavior
When the person is a leader, others follow what he/she does. If a person who has great prestige and status in the team hides knowledge, everyone will learn from him/her. This influence may have a greater impact on people in the same status or people whose statuses are lower than yours. Otherwise, the impact on people with higher statuses may not be great, because they do not care about you at all.	Individual status
We need to cooperate closely at work, so that his/her hidden knowledge is not good for himself/herself. Our work is independent and based on each other. Sometimes I can understand why he/she hides knowledge, because if he/she does not maintain his/her knowledge, he/she will be replaced. It is absolutely wise to hide knowledge in interdependent work.	Work interdependence
If you want to live well in a team, you should adapt to the team’s rules of operation. If you do not know how to do it, follow the majority. It is absolutely right to follow what most people do.	Herd mentality
I will learn from my colleagues in the same group. I’ve been learning from others. Whatever others do, I will do the same. I will imitate what the leader does.	Imitative learning
When personal interests conflict with team interests, I am willing to sacrifice personal interests. As a team member, I always consider the interests of the team in my work. Although it is called a team, we mostly still think based on individuals.	Collectivist orientation
I think I am an important part of our team. I am willing to work hard for the success of the team. The honor of the team is my honor.	Team identification
Sometimes this behavior mainly depends on whether the leader can manage. Our leaders give warnings or show punishment in some ways. What and how leaders do are the most important things.	Leaders’ supervision
Our team is assessed every year, and it pays more attention to the team members’ self-growth during the assessment. It also depends on how the team evaluates, whether it is developmental or evaluative. The assessment is according to results. If you published a good article and was granted a good patent for the team, you would be successful this year; otherwise, your performance would be poor.	Purpose of assessment
It creates an atmosphere of bad knowledge sharing. Conceivably, everyone does not share knowledge, and all the members feel that the overall state of knowledge sharing is bad. It is the overall atmosphere that if the team engages in knowledge hiding, no one would like to share it.	Poor knowledge sharing atmosphere
If one person hides, the supply of knowledge decreases. From the perspective of the process of knowledge sharing, no one provides knowledge and how to share it. Knowledge is resources and hiding knowledge indicates no resources. If there are no resources, how can we talk about knowledge exchange?	Reduced knowledge supply
Hiding knowledge is likely to be discovered, which is likely to affect mutual trust. Sometimes the member who hides knowledge is afraid of being discovered by others, which affects the relationship, mutual trust, and his/her reputation. Collective knowledge hiding in the team is likely to lead to collective distrust.	Interpersonal distrust
I share my knowledge selflessly in the team. Knowledge sharing is an important way to promote the development of a scientific research team. Our team regularly carries out knowledge sharing in the form of academic discussion meetings.	Knowledge sharing

It is noteworthy that unlike the general idea of data analysis, this study followed the general data analysis strategy of grounded theory research; thus, the analysis was carried out quickly after data collection, so that on the one hand, the understanding error during analysis can be smaller and the analysis can truly reflect the interviewees’ thoughts. On the other hand, it can also provide support for the next cycle of data collection and analysis. Finally, after completing the interviews with the 31 members of the scientific research team, there were no more new concepts and relations among the concepts, and the theory was essentially saturated.

### Axial Coding

After the open coding is completed, the study enters the axial coding stage. Axial coding is a process of organizing related categories around an “axis,” and it is also a process of deepening the cognition of scattered categories (Kendall, 1999). The primary goal in this stage is to develop the theory comprehensively. In order to achieve this goal, all categories were reorganized based on conceptual levels, dimensions, and characteristics through self-questioning. Moreover, through the common paradigm model of axial coding, the correlation between the various categories was analyzed, and the category levels, dimensions, and features were then classified. The classification results are presented in Table 3.

**TABLE 3:** Results of axial coding.

Main category	Subcategory	The connotation of category relations
Scientific research team’s knowledge hiding behavior	Individual knowledge hiding behavior	Individual knowledge hiding behavior is used to describe the knowledge hiding state of an individual member in the scientific research team, which is affected by the team’s collective knowledge hiding behavior and also affects the collective knowledge hiding behavior.
	Collective knowledge hiding behavior	The collective knowledge hiding behavior represents the overall state of a team’s knowledge hiding behavior, which is affected by the team’s individual knowledge hiding behavior and also affects individual knowledge hiding behavior.
Influence factors that influence the individual knowledge hiding behaviors transmitted to collective knowledge hiding behavior	Individual status	Individual status is an important factor influencing the effectiveness of individual knowledge hiding behavior transmitted to collective knowledge hiding behavior.
	Work interdependence	Work interdependence is also an important factor affecting the effectiveness of individual knowledge hiding behavior transmitted to collective knowledge hiding behavior.
Influence factors that influence collective knowledge hiding behavior transmitted to individual knowledge hiding behavior	Herd mentality	Herd mentality is an important mediating factor affecting collective knowledge hiding behavior transmitted to individual knowledge hiding behavior.
	Imitative learning	Imitative learning is an important mediating factor impacting collective knowledge hiding behavior transmitted to individual knowledge hiding behavior.
	Collectivist orientation	Collectivism orientation is an important moderating factor affecting collective knowledge hiding behavior transmitted to individual knowledge hiding behavior.
	Team identification	Team identification is an important moderating factor affecting collective knowledge hiding behavior transmitted to individual knowledge hiding behavior.
Influence factors of the knowledge hiding behavior interaction of scientific research teams	Leaders’ supervision	Leaders’ supervision is an important intervention factor for the interaction of knowledge hiding behavior in scientific research teams, which affects the entire process of knowledge hiding behavior interaction.
	Purpose of assessment	The purpose of assessment is an important intervention factor for the interaction of knowledge hiding behaviors in scientific research teams, which also influences the entire process of knowledge hiding behavior interactions. Different purpose of assessment may have different impacts.
Influence factors that influence the relation between the scientific research team’s knowledge hiding behavior interaction and knowledge sharing	Poor knowledge sharing atmosphere	The interaction of a scientific research team’s knowledge hiding behavior causes a poor knowledge sharing atmosphere in the scientific research team.
	Reduced knowledge supply	The interaction of a scientific team’s knowledge hiding behavior reduces the supply of the scientific team’s knowledge.
	Interpersonal distrust	The interaction of a scientific team’s knowledge hiding behaviors leads to an increase in the interpersonal distrust of the scientific team.
Knowledge sharing	–	Knowledge sharing is also an important category in this study, which is affected by knowledge hiding behavior.

(1) The formation of the main category of knowledge hiding behavior of the scientific research team: The interview data show that there are two types of knowledge hiding behaviors observed during the knowledge hiding behavior cross-level interaction within scientific research teams. One is for the individual members, namely, individual knowledge hiding behavior. For example, the interview data show, “Someone did this, that is to say he/she would hide what he/she knew, so I would learn from him/her.” The second is the collective knowledge hiding behavior of the team. Collective knowledge hiding behavior refers to the total amount of knowledge hiding that occurs within the team that is relevant, rather than the dispersion or variability in the hiding that occurs (Černe et al., 2015). The interview data, “When everyone hides knowledge, we basically form a culture, and an individual behavior becomes a collective behavior,” also indicate that individual knowledge hiding behavior is the inducement of collective knowledge hiding behavior, which often leads to the diffusion of individual knowledge hiding to collective knowledge hiding. Conversely, when collective knowledge hiding behavior appears, members in the team may consciously follow the overall norms of the team, thereby strengthening or promoting the generation of individual knowledge hiding behaviors.

(2) The formation of the category of influence factors that influence individual knowledge hiding behavior transmitted to collective knowledge hiding behavior: The interview data show that individual knowledge hiding behavior is transferred to collective knowledge hiding behavior, and this transmission is often affected by the status of the individual and the interdependence of the team work. For example, the interviewee said, “If a person who has great prestige and status in the team hides knowledge, everyone will learn from him/her.” Another interviewee said, “Our work is independent and based on each other. Sometimes I can understand why he/she hides knowledge because if he/she does not maintain his/her knowledge, he/she will be replaced.”

(3) The formation of the category of influence factors that influence collective knowledge hiding behavior transmitted to individual knowledge hiding behavior: the interview data also show that collective knowledge hiding behavior is transmitted to individual knowledge hiding behavior, and this transmission is often affected by four influencing factors, namely, herd mentality, imitative learning, collectivism orientation, and team identification. For example, one interviewee said, “It is absolutely right to follow what most people do.” Another interviewee indicated the importance of imitative learning: “I’ve been learning from others. Whatever others do, I will do the same.” Each factor plays a significantly different role, among which imitative learning and herd mentality play a mediating role in collective knowledge hiding behaviors transmitted to individual knowledge hiding behaviors; however, collectivism orientation and team identification play a moderating role.

(4) The formation of the category of interactive influence factors for the knowledge hiding behavior of the scientific research team: The interview data show that the scientific research team’s knowledge hiding behavior is a two-stage model that includes the individual knowledge hiding behavior transmitted to the collective knowledge hiding behavior and the collective knowledge hiding behavior transmitted to the individual knowledge hiding behavior. In the two-stage model, each has its own influencing factors. Moreover, there are also some factors that can directly affect the two stages. The interview data reveal that leaders’ supervision and the purpose of assessment are the most important factors influencing the social interaction of knowledge hiding behaviors in scientific research teams. For example, the interviewees say, “Our leaders give warnings or show punishment in some ways,” and “The assessment is according to results. If you published a good article and was granted a good patent for the team, you would be successful this year, otherwise your performance would be poor.”

(5) The formation of the categories of influence factors that influence the relationship between the scientific research team’s knowledge hiding behavior interaction and knowledge sharing: interview materials indicate that the scientific knowledge team’s knowledge hiding behavior interaction can negatively impact knowledge sharing. The impact mainly works through the following three aspects, namely, the poor knowledge sharing atmosphere (interview materials: *Conceivably, everyone does not share knowledge, and all the members feel that the overall state of knowledge sharing is bad*), reduction of the supply of *knowledge* (interview materials: *Knowledge is resources and hiding knowledge indicates no resources. If there are no resources, how can we talk about knowledge exchange?*), and interpersonal distrust (interview material: *Collective knowledge hiding in the team* is likely to *lead to collective distrust*).

(6) Knowledge sharing: the interview data show that knowledge sharing is also an important concept in this study. Through the analysis of interview data, we found that there is no other concept that can be classified into a category with knowledge sharing; thus, we regard it as an independent category.

### Selective Coding

Selective coding is the process of systematically collating qualitative data and information and realizing theoretical construction and development. Moreover, it is also the ultimate foothold for considering the data analysis of grounded theory (Hernandez, 2009). Its main objective is to sort out the scattered main categories, discover the relationship between the main categories, further condense the core categories of research, and finally construct a complete theoretical process around the core categories. When grounded theory is utilized for analysis, the selection of core categories often has its own specific criteria. In order to determine the core category of this research, we again reviewed the research results of open coding and axial coding, and we kept asking the following questions: “What is the relationship between the main categories?”; “Which problem is the core of the research that is at the center position of the materials?”; “Which problem can provide the abstract expression of all main and sub-categories?”; and “Which category can be changed without changing the interviewees?” In the process of looking for the answers to these four questions by studying the interview records, the authors gradually discovered and clarified the core category of this research: “The interactive mechanism of knowledge hiding behaviors in scientific research teams between individual knowledge hiding behavior and collective knowledge hiding behavior and its impact on knowledge sharing.”

**FIGURE 1:**
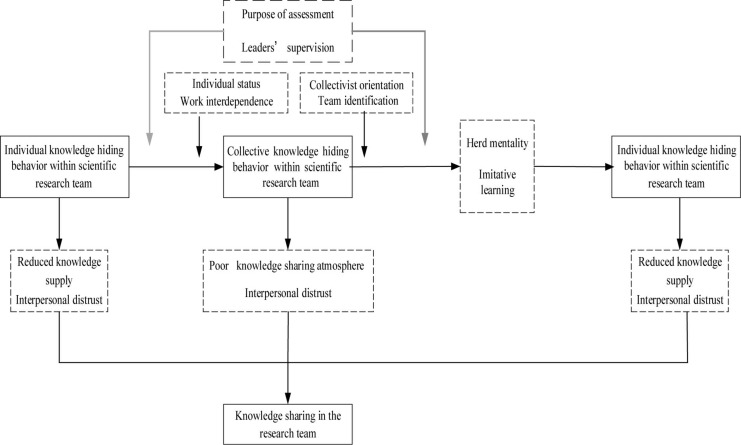
Theoretical model of a scientific research team’s knowledge hiding behavior interaction and its impact on knowledge sharing.

After the core category was determined, it was necessary to describe and depict the complex relationship between various categories in the form of a “story line” (Corbin and Strauss, 1990; Strauss and Corbin, 1997). The process of the complete description and depiction of a “story” is the process of final theoretical development. The relationship between the various categories is presented in Table 4 and Figure 1.

**TABLE 4:** Results of selective coding.

Typical relationship structure	Connotation of relational structure
Individual knowledge hiding → collective knowledge hiding	Individual knowledge hiding behavior of scientific research team members can affect collective knowledge hiding behavior.
Collective knowledge hiding → individual knowledge hiding	Collective knowledge hiding behavior of a scientific research team can affect individual knowledge hiding behavior.
Individual status and work interdependence ↓ Individual knowledge hiding → collective knowledge hiding	Individual status and work interdependence affect the relationship between individual knowledge hiding behavior and collective knowledge hiding behavior within scientific research team members.
Collective knowledge hiding → imitative learning and herd mentality → individual knowledge hiding	Imitative learning and herd mentality serve as a bridge and a mediator between the collective knowledge hiding behavior and individual knowledge hiding behavior within a scientific research team.
Collectivist orientation and team identification ↓ Collective knowledge hiding → imitative learning and herd mentality	Collectivist orientation and team identification affect the relationship between collective knowledge hiding behaviors and imitative learning as well as herd mentality.
Leaders’ supervision and assessment purposes ↓ ↓ Individual knowledge hiding → collective knowledge hiding → individual knowledge hiding	The purpose assessment and leaders’ supervision affect the interactive mechanism of knowledge hiding behaviors within a scientific research team.
Individual knowledge hiding → collective knowledge hiding → individual knowledge hiding ↓ Interpersonal distrust, reduction of knowledge supply, and poor knowledge sharing atmosphere ↓ Knowledge sharing	Knowledge hiding behavior interaction can affect sustainable knowledge sharing. Interpersonal distrust, reduction of knowledge supply, and a poor knowledge sharing atmosphere play a mediating role.
	

## Discussion

By integrating the research process and conclusion of the steps of the axis coding and selective coding mentioned above, we further tried to integrate the specific pairs of selective coding to build a conceptual model of the interactive mechanism of knowledge hiding behaviors in scientific research teams and its impact on knowledge sharing (see Figure 1).

This study indicates that the scientific research team’s knowledge hiding behavior interaction is a two-stage interaction model. The first stage is “the interaction of the scientific research team’s individual knowledge hiding behavior and collective knowledge hiding behavior.” In this stage, the individual’s knowledge hiding behavior spreads to the collective knowledge hiding behavior, and the effectiveness of the diffusion is affected by individual status and work interdependence. Our interview record supports this view: “When everyone hides knowledge, we basically form a culture, and an individual behavior becomes a collective behavior.” This is also similar to the related research on the “bad apple” effect by related scholars (Gino et al., 2009). Our study also indicates that if the status of an individual with knowledge concealment is higher, the behavior is more likely to be imitated by team members; in contrast, the impact will be smaller, which is similar to the study of status or power. Our interview record also supports this view: “If a person who has great prestige and status in the team hides knowledge, everyone will learn from him/her.” This is consistent with the theoretical assumption of knowledge as power (Mudambi and Navarra, 2004). Moreover, our research also finds that work interdependence also influences the excessive transfer of individual knowledge hiding behavior to collective knowledge hiding behavior, which can be supported by the interviewee. For example, one interviewee said, “Our work is independent and based on each other. Sometimes I can understand why he/she hides knowledge because if he/she does not maintain his/her knowledge, he/she will be replaced.” This is in line with the general assumptions of social learning theory (Bandura, 1977) and social interaction theory (Ben-Sira, 1976).

The second stage is the “interaction between the collective knowledge hiding behavior and the individual knowledge hiding behavior of the scientific research team.” At this stage, the knowledge hiding behavior of the scientific research team is also transmitted to the individual, which is consistent with the behavior of peer influence discussed by related scholars in the field of organizational behavior (Gino et al., 2009). The study also found that this contagious process is mediated by herd mentality and imitative learning. Herd mentality is an important concept in the field of psychological research (Vishwanath and Scamurra, 2007). Team members with herd mentality are more likely to accept team-level influences. Our interview record supports this view: “If you do not know how to do it, follow the majority.” However, imitative learning is an important way for team members to learn organizational behaviors. The conclusions of the study are consistent with the relevant results of the social learning theory. Furthermore, interviewees also indicated that the mediating role of herd mentality and imitative learning is also affected by collectivist orientation and team identification. The more obvious the collectivist orientation, the more the team identification, the stronger their relationship, and vice versa.

Moreover, the study also found that the magnitude of the effect of the two stages is affected by the leaders’ supervision and purpose of assessment. The interview data indicate that leaders’ supervision is likely to cut off the influence of the individual knowledge hiding behavior on the collective knowledge hiding behavior, and it can also cut off the influence of the collective knowledge hiding behavior on the individual knowledge hiding behavior. This result is consistent with previous research conclusions on unethical behavior and organizational behaviors, such as corruption. Furthermore, the objective of different types of assessments can also prevent or promote the interaction of cross-level knowledge hiding behavior. This also shows inner consistency in the conclusions of scholars referring to the relationship between knowledge hiding behavior and creativity (Černe et al., 2014).

The study found that the knowledge hiding behavior interaction has a negative impact on knowledge sharing. The impact is reflected in the following three factors. First, under the influence of the “bad apple” when the individual knowledge hiding behavior occurs in a scientific research team, the first manifestation is the reduction of individual knowledge supply, which is consistent with the findings of research on social exchange theory; that is, the most important reason for the failure of knowledge sharing is insufficient knowledge supply. This view is supported by the interviewee. For example, an interviewee said, “Knowledge is resources, and hiding knowledge indicates no resources. If there are no resources, how can we talk about knowledge exchange?” Second, interpersonal distrust is another important factor affecting the relationship between knowledge hiding behavior and knowledge sharing, which is supported by the interview record: “Sometimes the member who hides knowledge is afraid of being discovered by others, which affects the relationship, mutual trust, and his/her reputation.” This result is similar to the research findings of Connelly et al. (2012). That is, knowledge hiding behavior generates a state of interpersonal distrust and further affects knowledge exchange among members and leads to the failure of knowledge sharing. In addition, poor knowledge sharing atmosphere is a third important factor. In other words, a scientific research team with a high level of team knowledge hiding behaviors naturally creates a poor knowledge sharing atmosphere. When a poor knowledge sharing atmosphere is created within a team, knowledge sharing is difficult to sustain. Our interview records support this view, such as the following: “Conceivably, everyone does not share knowledge, and all the members feel that the overall state of knowledge sharing is bad”.

### Theoretical Contributions and Practical Implications

This research makes a significant contribution to the existing studies on knowledge hiding behavior by constructing a cross-level interactive cycle model between individual knowledge hiding behavior and collective knowledge hiding behavior within the scientific research team. Current studies show that knowledge hiding behavior not only occurs at the dyadic level but also occurs in teams and forms collective-level knowledge hiding behavior (Černe et al., 2015). However, to date, research on the interactive relationship between individual-level knowledge hiding behavior and collective-level knowledge hiding behavior is still limited (Connelly et al., 2019). Therefore, this research attempts to fill this gap. Our research shows that individual knowledge hiding behavior in scientific research teams will promote the formation of knowledge hiding behavior at the collective level, and its role is mainly affected by the individual status of the knowledge hiding person and the work interdependence within the team; that is, the higher the status of the implementer of knowledge hiding behavior, the easier it is for individual knowledge hiding behavior to form collective knowledge hiding behavior. Different from the role of individual status, the interview data show the influence of work interdependence on two different directions between individual and collective knowledge hiding. Similarly, collective knowledge hiding behavior will also promote the formation of individual knowledge hiding behavior, and this role is mainly played through the mediating effect of herd mentality and imitative learning. In addition, the research results show that the role of herd mentality and imitative learning will be affected by collectivism orientation and team identification; thus, the higher the collectivism orientation and team identification of individuals in the team, the more vulnerable individuals are to collective knowledge hiding behavior, thereby producing individual knowledge hiding behavior. Moreover, we have also identified two important influencing factors of the cross-level interaction cycle of individual knowledge hiding behavior and collective knowledge hiding behavior, that is, leaders’ supervision and the purpose of assessment.

This research is also helpful in understanding the relationship between different levels of knowledge hiding behavior and sustainable knowledge sharing within the research team. Current studies have fully demonstrated the importance of knowledge sharing and extensively discussed the antecedents of knowledge sharing (Anand and Jain, 2014; Pan et al., 2018; Hernaus et al., 2019). Among these antecedents, scholars have noticed that knowledge hiding behavior weakens the sustainable knowledge sharing behavior within the team and verified the important role of distrust, which is consistent with our research conclusions (Connelly et al., 2012); that is, interpersonal distrust is an important factor affecting the relationship between knowledge hiding behavior and knowledge sharing. In addition, we have also identified another two important factors that affect knowledge hiding behavior and knowledge sharing; that is, knowledge hiding behavior affects knowledge sharing within the team by reducing knowledge supply and forming a poor knowledge sharing atmosphere. These research findings will deepen our understanding of the antecedents of knowledge sharing as well as the relationship between knowledge hiding behavior and knowledge sharing.

Our study also has several implications for scientific research team managers. First, the research finds that individual knowledge hiding behavior will promote the formation of collective knowledge hiding behavior. Therefore, when individual knowledge hiding behavior takes place in teams, it is necessary to intervene in the individual knowledge hiding behavior in time to hinder the formation of collective knowledge hiding behavior. In addition, compared with individuals with low team status, scientific research team managers also need to pay more attention to knowledge hiding individuals with higher team status. Second, the study found that collective knowledge hiding behavior will affect individual knowledge hiding behavior. This effect depends on the imitative learning and herd mentality of individual team members. Further research also found that collectivist orientation and team identification are key regulatory factors that affect imitative learning and herd mentality. Therefore, when a team is in a state of high collective knowledge hiding behavior, team managers need to take diversified measures to cultivate the individuality of team members; create a good atmosphere of innovation and fault tolerance and thereby weaken the collective orientation, team identification, imitative learning, and herd mentality; and ultimately prevent the spread of collective knowledge hiding behavior to individual knowledge hiding behavior. Third, our research also found that the leader’s supervision and the purpose of assessment are important factors that affect the cross-level interaction cycle of individual knowledge hiding behavior and collective knowledge hiding behavior. Therefore, managers of scientific research teams should be mindful of knowledge hiding behavior to timely discover, intervene, and take charge of knowledge hiding behavior. Furthermore, scientific research team managers should also set a reasonable purpose for assessment. Through adjustments to the purpose of assessment, scientific research team managers could hinder the cross-level interaction between individual knowledge hiding behavior and collective knowledge hiding behavior in a timely manner. Finally, the research results show that knowledge hiding behavior mainly weakens sustainable knowledge sharing within the team by reducing knowledge supply, forming interpersonal distrust, and establishing a poor knowledge sharing atmosphere. Therefore, team managers need to formulate good knowledge sharing incentive policies to increase the supply of knowledge and reduce the impact of knowledge hiding on knowledge sharing. In addition, team managers should make efforts to establish trust relationships within the scientific research team. Managers also need to take measures to build effective knowledge sharing policies and constitute knowledge sharing information systems in order to promote the team to form a good knowledge sharing atmosphere and further weaken the influence of knowledge hiding behavior on sustainable knowledge sharing.

### Limitations and Suggestions for Future Research

Undeniably, this article has certain limitations. First, this study does not focus on the knowledge interaction between different individuals or different scientific research teams during the implementation of knowledge hiding behavior. This is mainly attributed to the fact that this study mainly explores from the perspective of the individual-collective interaction. Nonetheless, the interaction of individual-to-individual knowledge hiding behaviors exists, and related research studies have been conducted by Connelly et al. (2012) and Connelly and Zweig (2015). Moreover, the research samples in this study are mainly based on Chinese scientific research teams, which have certain geographical limitations, and the distribution of samples can be further expanded in the future. Moreover, related scholars have pointed out that knowledge hiding behavior can be divided into different specific types, and the impact of different specific types of knowledge hiding behaviors may vary; however, the differences among specific knowledge hiding behaviors were not explored herein. More explorations can be conducted in the future to divide knowledge hiding behaviors into different types and to explore the differences in the interaction mechanisms between different types of individual knowledge hiding behavior and collective knowledge hiding behavior and the differences in their impact on knowledge sharing. In addition, our findings and conclusions are mainly based on interview data and are still subjective compared to quantitative research. Therefore, it is necessary to conduct quantitative research in the future to increase the reliability and validity of the research results.

## Conclusion

Based on the research objectives, 31 representative members of 9 scientific research teams were selected to participate in semi-structured interviews to collect qualitative data on knowledge hiding behavior. Subsequently, the qualitative data were obtained through three necessary steps, namely, open coding, axis coding, and selective coding, to gradually reveal the interactive process of knowledge hiding behaviors in scientific research teams between individual knowledge hiding behavior and collective knowledge hiding behavior and to clarify the complex influence on knowledge sharing. The results show that the scientific research team’s knowledge hiding behavior interaction is a two-stage cross-level interaction model. The effect of the first stage is influenced by individual status and work interdependence; however, the effect of the second stage is influenced by herd mentality, imitative learning, collectivism orientation, and team identification. Furthermore, the study also found that the purpose of assessment and leaders’ supervision affect both phases simultaneously, resulting in effective intervention or inhibition. Additionally, the results also revealed that knowledge hiding behavior can affect the sustainable knowledge sharing of the research team by reducing the supply of knowledge, creating a poor knowledge sharing atmosphere, and forming an interpersonal distrust relationship. This research can also provide a basis for a deeper understanding of the interaction mechanism of knowledge hiding behavior and its impact on knowledge sharing.

## Data Availability Statement

The raw data supporting the conclusions of this article will be made available by the authors, without undue reservation, to any qualified researcher.

## Ethics Statement

This study was approved by the Institutional Review Board of Institute of China Innovation and Entrepreneurship Education at Wenzhou Medical University and each participant signed the informed written consent.

## Author Contributions

FL contributed to research design, data analysis, and manuscript writing. YL provided efforts on research design. PW contributed to research design and manuscript writing and provided quality assurance of the research. All authors contributed to the article and approved the submitted version.

## Conflict of Interest

The authors declare that the research was conducted in the absence of any commercial or financial relationships that could be construed as a potential conflict of interest.
